# Improving Drug Uptake and Penetration into Tumors: Current and Forthcoming Opportunities

**DOI:** 10.3389/fonc.2013.00161

**Published:** 2013-06-18

**Authors:** Fabrizio Marcucci, Ronald Berenson, Angelo Corti

**Affiliations:** ^1^Centro Nazionale di Epidemiologia, Sorveglianza e Promozione della Salute, Istituto Superiore di Sanita’Roma, Italy; ^2^Hepatology Association of CalabriaReggio Calabria, Italy; ^3^ImmunoMod ConsultingMercer Island, WA, USA; ^4^Tumor Biology and Vascular Targeting Unit, Division of Molecular Oncology, San Raffaele Scientific InstituteMilano, Italy

The main scope of this topic is to give an update on approaches being studied and developed to improve tumor drug delivery through active targeting and other methods. Inadequate drug accumulation has emerged as one of the main problems underlying therapeutic failure and drug resistance in the treatment of solid tumors (Trédan et al., 2007; Marcucci and Corti, 2012a). It is causally related to the abnormal tumor architecture. Poor vascularization, increased resistance to blood flow and impaired blood supply represent a first obstacle to the delivery of antitumor drugs to tumor cells. Decreased or even inverted transvascular pressure gradients compromise convective transport of drugs. Eventually, an abnormal extracellular matrix offers increased frictional resistance to tumor drug penetration. The net result is reduced overall drug accumulation in tumors, and the propensity of drugs to accumulate in perivascular spaces without penetrating vessel-distant tumor areas. This promotes passive and active induction of drug resistance (Marcucci and Corti, 2012b).

Abnormal tumor architecture, inadequate drug accumulation and tumor drug resistance are tightly linked phenomena, suggesting that normalization of the tumor architecture, including tumor blood vessels, may result in increased drug delivery to tumors and improve the therapeutic efficacy of anticancer drugs. Indeed, several classes of drugs, that we have referred to as promoter drugs, (Marcucci and Corti, 2012a) have been reported to increase tumor uptake and penetration of antitumor drugs, including drugs that are: (1) vasoactive (Nagamitsu et al., 2009), (2) normalize tumor vessels (Jain, 2005), (3) modify the barrier function of tumor vessels (Corti and Marcucci, 1998; Curnis et al., 2000, 2002), (4) debulk tumor cells (Padera et al., 2004; Moschetta et al., 2012), (5) overcome intercellular (Beyer et al., 2011, 2012; Wang et al., 2011) and stromal barriers (Provenzano et al., 2012). In addition, non-pharmacologic approaches have been described that enhance tumor accumulation of effector drugs (e.g., convection-enhanced delivery, hyperthermia, ultrasound, etc.) (Sen et al., 2011; Watson et al., 2012).

Some drugs that have already received regulatory approval (e.g., the anti-vascular endothelial growth factor antibody bevacizumab) (Hurwitz et al., 2004) exert antitumor effects at least in part by normalizing the tumor vasculature and enhancing tumor accumulation of chemotherapeutic drugs (Willett et al., 2004). Bevacizumab, however, has a problematic side-effect profile, and the effective doses of the drug encompass a very narrow range beyond which it may even lead to a reduction in drug delivery (Van der Veldt et al., 2012). Additional drugs, acting through other mechanisms of action, are now in clinical development (e.g., vascular targeted NGR-tumor necrosis factor, in phase II/III studies) (Sacchi et al., 2006) and others are about to enter clinical investigation (e.g., Junction Opener-1) (Beyer et al., 2011, 2012).

To date, the focus has been primarily on the identification of novel promoter drugs that improve tumor drug delivery. This has led to a considerable number of promoter drugs and devices that are effective in preclinical studies, and some of which have proceeded into clinical investigation or are about to do so. Regarding the types of drugs to be delivered, chemotherapeutics have been the obvious first choice, because they are the antitumor drugs in most widespread use (Curnis et al., 2002; Beyer et al., 2012). Another area of interest is antitumor monoclonal antibodies or related compounds (e.g., immunocytokines) (Beyer et al., 2011; Moschetta et al., 2012), which have become an important component of the antitumor drug armamentarium over the last 15 years. Preclinical investigations have produced promising results when these therapeutic agents are combined with drugs that enhance their penetration into tumors, and it is reasonable to predict that clinical studies will follow in the forthcoming years. So far, so good, but what next? Have we looked at all possible applications for promoter drugs, or are there further applications that we can envisage? We believe that there is still an important field of application for promoter drugs that has been relatively unexplored so far, i.e., the possibility to improve delivery of anticancer cells, in particular immune cells to the tumor (Marcucci et al., 2013), an area of increasing clinical interest.

Enhancing penetration of immune cells into tumors may have two main therapeutic applications. The first is to improve the efficacy of immune-regulatory antibodies, such as the anti-cytotoxic T-lymphocyte antigen-4 antibody ipilimumab, and the anti-programed death-1 antibody nivolumab. These antibodies yield impressive, and often long-lasting therapeutic responses in a limited fraction (10–20% depending on the antibody) of heavily pretreated patients with metastatic melanoma and other solid tumors (Hodi et al., 2010; Topalian et al., 2012). There is a relationship between the number of tumor-infiltrating immune cells and responsiveness to ipilimumab (Lynch et al., 2012). In this setting, promoter drugs could be of value at two levels: first, to improve tumor delivery of the antibody itself, and second, improve penetration of immune cells into the tumor. This has the potential to increase the fraction of patients that become responders to these antibodies. A second possible field of application are antitumor vaccines. Antitumor vaccines are often active only when administered in a prophylactic setting. With growing tumors, vaccination becomes progressively less effective. One reason might be that tumor-specific lymphocytes become sensitized in draining lymph nodes but are then unable to enter tumors and eliminate tumor cells (Ganss and Hanahan, 1998). Promoter drugs that improve infiltration of immune cells into tumors may prove useful in increasing the effectiveness of cancer vaccines. However, infiltration of immune cells into tumors has requirements that go beyond those of antitumor drugs. Physiological pathways of immune cell extravasation depend on a multistep cascade of events involving tethering, rolling, firm adhesion, and migration. These steps are mediated by distinct adhesion molecules and activation pathways (Springer, 1994); however, adhesion molecules are often downregulated on tumor endothelial cells, a phenomenon defined as endothelial cell anergy (Piali et al., 1995). This impairs the entry of immune cells into tumor sites. In order to enhance tumor infiltration of immune cells, promoter drugs may be required that induce a local inflammatory reaction. This leads to up-regulation of adhesion receptors that are able to attach immune cells to vessel walls and enable their penetration into tumors. Preliminary studies suggest that certain promoter drugs may achieve this goal (Calcinotto et al., 2012).

Promoter drugs that improve tumor delivery of chemotherapeutics and antitumor antibodies are likely to become a clinical reality in forthcoming years. In addition, new possibilities are emerging to enhance the entrance of therapeutic agents into tumors. For example, recent results suggest that promoter drugs may be useful also for improving infiltration of immune cells into tumors. This may increase the antitumor effects of a broad range of immune-based therapeutics, including immune-regulatory antibodies, antibodies that engage cytotoxic immune cells, and cancer vaccines.
